# *Feline Calicivirus* P39 Inhibits Innate Immune Responses by Autophagic Degradation of *Retinoic Acid Inducible Gene I*

**DOI:** 10.3390/ijms24065254

**Published:** 2023-03-09

**Authors:** Jianwei Mao, Shaotang Ye, Jie Deng, Jie Song, Zhen Wang, Aolei Chen, Pei Zhou, Shoujun Li

**Affiliations:** 1 College of Veterinary Medicine, South China Agricultural University, Guangzhou 510642, China; 2 Guangdong Technological Engineering Research Center for Pet, Guangzhou 510642, China

**Keywords:** feline calicivirus, autophagy, RIG-I, P39, viral replication, innate immunity

## Abstract

*Feline calicivirus* (FCV) is a feline pathogen that can cause severe upper respiratory tract disease in cats, thus posing a major threat to their health. The exact pathogenic mechanism of FCV is still unclear, although it has been identified as having the ability to induce immune depression. In this study, we discovered that FCV infection triggers autophagy and that its non-structural proteins, P30, P32, and P39, are responsible for initiating this process. Additionally, we observed that altering autophagy levels via chemical modulation resulted in different influences on FCV replication. Moreover, our findings indicate that autophagy can modify the innate immunity induced by FCV infection, with increased autophagy further suppressing FCV-induced RIG-I signal transduction. This research provides insights into the mechanism of FCV replication and has the potential to aid in the development of autophagy-targeted drugs to inhibit or prevent FCV infection.

## 1. Introduction

Feline calicivirus (FCV), a single-stranded positive-sense RNA virus of the genus Vesivirus in the family *Caliciviridae*, was first identified in 1957 [1]. *Caliciviridae* includes important pathogens in humans, such as norovirus (Nov), which is one of the most common causes of human infectious gastroenteritis, and important pathogens in other animal species, such as rabbit hemorrhagic disease virus (RHDV) and rabbit calicivirus (RCV) [2]. FCV is capable of infecting all known felines, including leopards, lions, and tigers, and it has become an important health concern for both wild and domestic cats [3,4]. The FCV genome is approximately 7.7 kb long and encodes 6 non-structural proteins (VPg, P5.6, PP, P30, P32, and P39) and 2 structural proteins (VP1 and VP2) [5]. Recently, a virulent systemic disease (VSD) associated with FCV infection has been reported globally. This strain, known as FCV-VSD, can cause severe symptoms such as a high fever, edema, ulcerative limbs, and jaundice, and can result in a high mortality in adult cats [6,7,8]. After entering cells, FCV replicates rapidly and evades the innate immune monitoring. Some studies have pointed out that the mechanisms of FCV could evade the innate immune response by inactivating the IFN-β promoter [9], and the non-structural proteins p30, p32, and p33 of FCV lead to the reorganization of the endoplasmic reticulum membrane [10].

Autophagy, a lysosome-dependent degradation pathway found in eukaryotic cells, is a vital process for maintaining cellular equilibrium by removing damaged organelles, misfolded, and long-lived cytoplasmic proteins for recycling. Several internal and external stimuli, including viral infection, endoplasmic reticulum (ER) stress, cellular starvation, and impaired organelles, can activate autophagy. The relationship between the virus of the family *Caliciviridae* and autophagy has been widely studied. Murine norovirus (MNV) hijack host cellular autophagy to facilitate its own replication [11], and HuNoV cultured at 37 °C showed a significantly increased expression of autophagy-related genes [12]. Nonetheless, the correlation between FCV replication and autophagy has not yet been investigated.

The innate immune response is the first line of defense against invading viruses [13]. Meanwhile, many viruses have evolved a diverse range of strategies to evade host innate immune responses. FCV has been found to utilize a variety of strategies to evade the host innate immune response. Studies have demonstrated that the FCV proteinase–polymerase (PP) can reduce host mRNA expression and P30 can directly degrade IFNAR1 mRNA [14,15]. RIG-I, MDA5, and LGP2/DHX58 are pattern recognition proteins (PRRs) with the structural domain of DExD/H-box RNA helicase [16]. RIG-I is responsible for recognizing non-self RNA in the cytoplasm, such as viral RNA, which leads to the translocation of IRF-3 and NF-κB to induce the expression of type 1 interferon (IFN) and inflammatory cytokines, thereby mediating host antiviral immunity [17,18]. Additionally, RIG-I-mediated signaling can induce autophagy through the MAVS-TRAF6-Beclin1 signaling axis [19], and autophagy has been shown to regulate the activation of the RLRs pathway and expression of exogenous RIG-I [20]. Despite the progress made in understanding the pathogenesis and immune evasion strategies of FCV, the mechanisms of FCV replication and the host innate immune response remain largely unknown.

FCV is a highly contagious virus that causes respiratory and oral diseases in cats. While much research has been carried out to understand the pathogenic mechanisms of FCV, the relationship between FCV and autophagy, as well as the underlying mechanisms, remain unclear. In this study, we aimed to investigate the relationship between FCV infection and autophagy induction and to explore the mechanisms by which autophagy facilitates FCV replication. We also aimed to examine the relationship between autophagy induced by the non-structural proteins of FCV and the RIG-I signaling pathway. Our research aimed to elucidate a novel immune evasion mechanism of FCV that facilitates replication by inhibiting the RIG-I-mediated innate immune signaling pathway through autophagy.

## 2. Results

### 2.1. Feline Calicivirus Infection-Induced Autophagy

**Figure 1:**
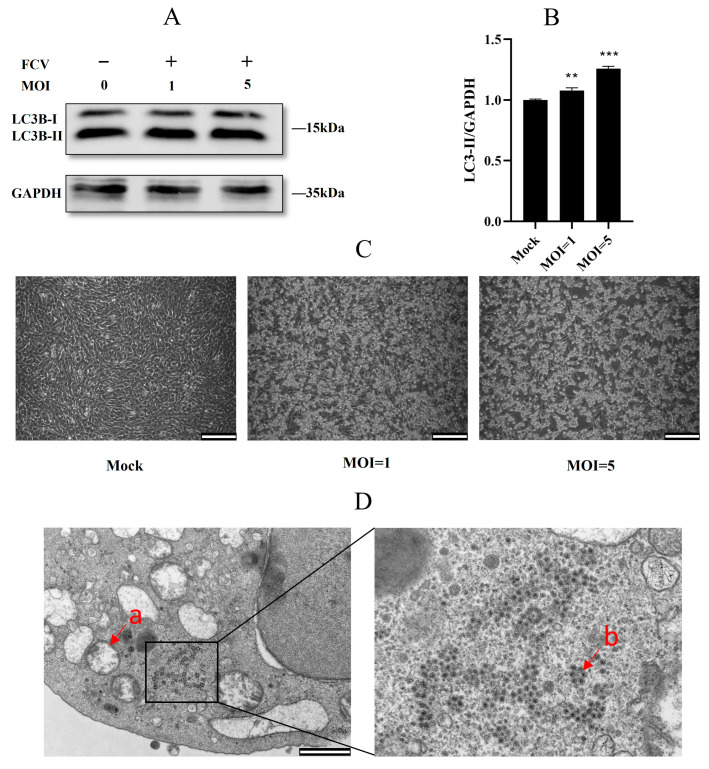
Feline calicivirus infection-induced autophagy. (**A**) With the increase in MOI, the expression of LC3-II was correspondingly increased. (**B**) Protein band intensity of LC3B-II and GAPDH. (**C**) A higher infection of MOI indicated more evident CPE. (**D**) CRFK cells were infected with FCV and fixed for resin embedding at 12 h.p.i. Double membrane vesicles can be observed in the cytoplasm of cells infected with FCV. The double membrane vesicles are identified with a, and FCV virions with b. Scale bar 1 um. (** *p* < 0.01, *** *p* < 0.001).

In order to determine whether FCV infection triggers cellular autophagy, we examined the protein expression of LC3B, an important marker of autophagy, in CRFK cells with different MOI. The Western blot analysis of LC3B-II/GADPH revealed a significantly higher relative ratio in FCV-infected cells, particularly in those exposed to a higher MOI (Figure 1A–C). To further validate the induction of autophagy by FCV, we performed transmission electron microscopy (TEM) analysis. Our TEM results demonstrated the presence of double-membrane structures of various sizes, including the classical autophagosome structure, as well as FCV particles in the same cell (Figure 1D). These findings collectively suggest that FCV infection can indeed initiate cellular autophagy in CRFK cells.

### 2.2. Non-Structural Protein P30, P32, and P39 Were Responsible for Autophagy Initiation

**Figure 2:**
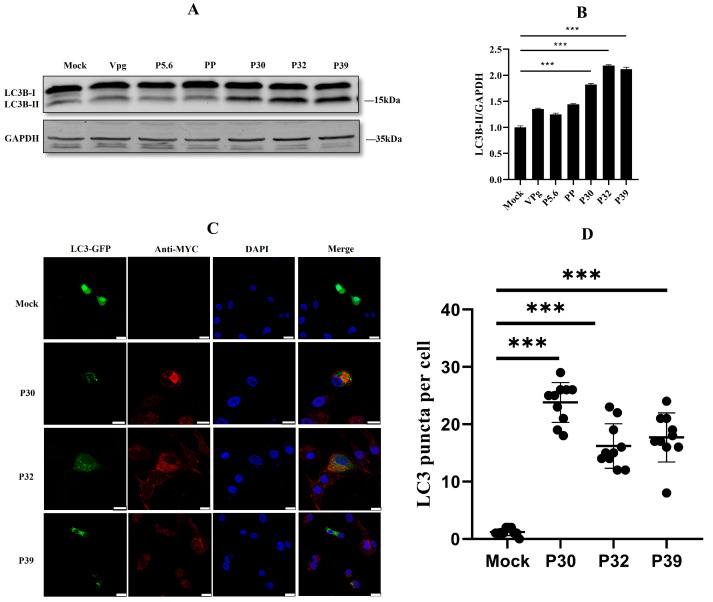
P30, P32, and P39 were responsible for autophagy initiation. (**A**) Excessive expression of FCV non-structural protein in the HEK 293T cell enhanced the expression of LC3B-II, and GAPDH was used as a loading control. (**B**) Protein band intensity of LC3B-II and GAPDH was used for Western blotting quantification analysis. Samples were analyzed in three independent experiments (*** *p* < 0.001). (**C**) HEK 293T cells were transfected with FCV non-structural protein (Myc-tagged) and GFP-LC3 (green). Scale bar 10 um. (**D**) Quantification of the number of GFP-LC3 puncta in mock-infected cells compared to FCV-infected at 12 h.p.i, error bars indicate the number of foci per cell ± SD and significance was determined by students *t*-test. (*** *p* < 0.001).

In the last part, we discovered that FCV infection induced autophagosome formation. This interesting phenomenon needed further investigations to unveil what viral proteins were responsible for autophagy initiation. A previous report noted that the non-structural proteins of FCV play an important role in the reorganization of ER membranes [10]. Here, we constructed six Myc-tagged non-structural proteins of FCV. These proteins were overexpressed in the HEK293T cells, and they were observed to quantify the autophagy process. The Western blotting results showed that P30, P32, and P39 could significantly change the ratio of LC3B-II/GAPDH (Figure 2A,B). Moreover, in confocal microscopy observation, the increasing puncta numbers of GFP-LC3 were captured in the overexpression of FCV P30, P32, and P39 proteins in HEK293T cells (Figure 2C,D). These results suggested that FCV P30, P32, and P39 were the main causes of autophagy induction.

### 2.3. Autophagy Promoted Feline Calicivirus Replication

**Figure 3:**
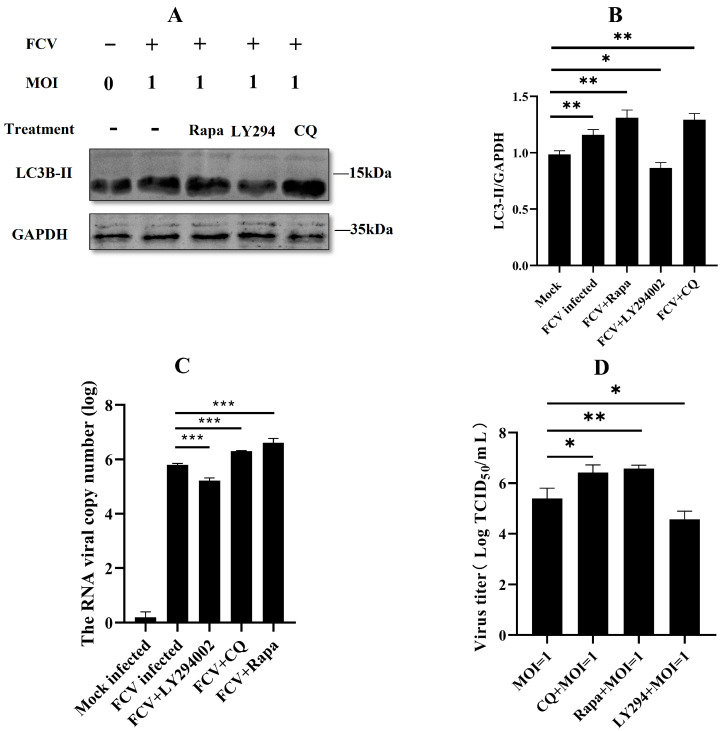
Autophagy promoted feline calicivirus replication. (**A**) Cells were treated with rapamycin (1 µM), ly294002 (20 µM), and chloroquine (50 µM) for 24 h, then CRFK cells were infected with FCV-SCAU-10. Protein band intensity of LC3B-II and GAPDH was used for Western blotting quantification analysis. Samples were analyzed in three independent experiments. (**B**) Protein band intensity of LC3B-II and GAPDH. (**C**,**D**) Supernatants were collected and subjected to TCID50 and qPCR. (* *p* < 0.05, ** *p* < 0.01, *** *p* < 0.001).

Our early investigation showed that FCV infection induced autophagy. However, we did not know which functions of autophagy serve FCV infection. So, we treated the cells with different drugs (rapamycin, ly294002, and chloroquine). Overall, the three compounds—chloroquine, LY294002, and rapamycin—each have distinct mechanisms of action that result in their differing effects on cellular autophagy. Chloroquine exerts its inhibitory effect by interfering with the lysosomal function, which disrupts the normal degradation of cellular components. LY294002, a small molecule inhibitor of the phosphatidylinositol 3-kinase (PI3K) pathway, relieves the negative regulation of autophagy by inhibiting the Akt/mTOR pathway, which is normally activated by PI3K. In contrast, rapamycin promotes autophagy by inhibiting the mammalian target of the rapamycin (mTOR) pathway, which is a major negative regulator of autophagy. By specifically inhibiting mTOR, rapamycin activates the Unc-51-like kinase 1 (ULK1) complex, which is necessary for the initiation of autophagy. Thus, while each of these compounds has the potential to modulate autophagy, they do so through distinct mechanisms that have different effects on the autophagic process (Figure 3A,B). Then, the pre-treated CRFK cells were infected by FCV, and the supernatant was harvested 12 h after virus inoculation. The supernatant of infected cells was used for the quantification of virus replication. The virus RNA and virus titer results showed that the FCV could significantly benefit from the facilitation of autophagy and the inhibition of late autophagy, while the inhibition of early autophagy dampened the FCV replication (Figure 3C,D). These results suggested that autophagy may play an essential role in the FCV replication cycle.

**Figure 4:**
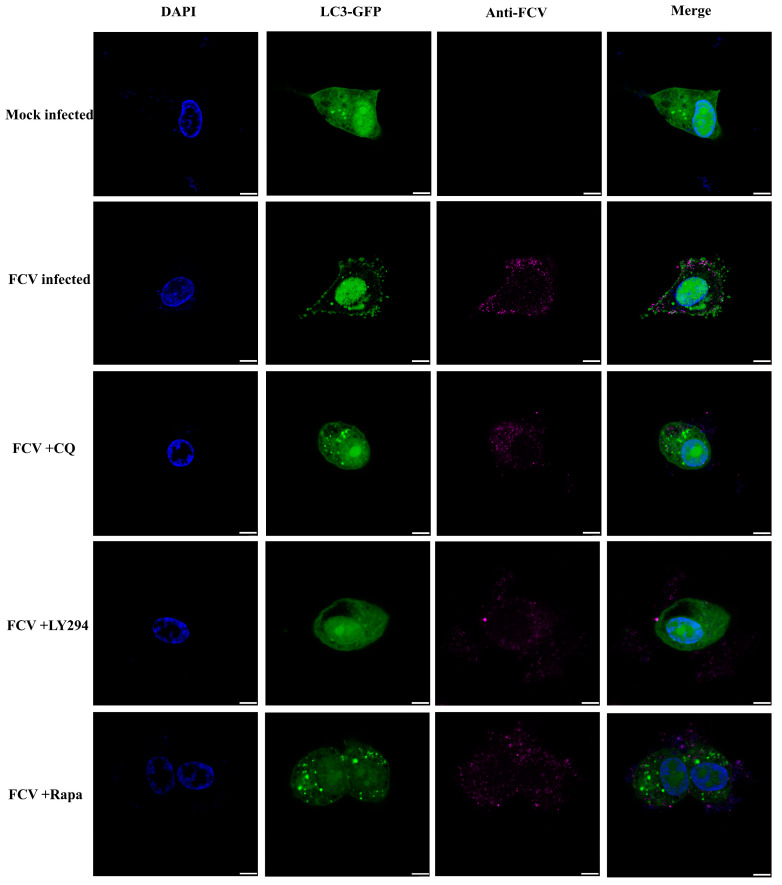
CRFK cells were transfected with GFP-LC3 (green), and 24 h later, infected with FCV at an MOI of 1, fixed at 12 h.p.i., and labeled with FCV antibody (purple). Scale bar 5 μm.

To further investigate the role of FCV infection in autophagy modulation, we conducted confocal experiments following drug treatment and FCV infection. Interestingly, we observed a significant increase in GFP-LC3 punctate aggregation in the FCV-infected group, as well as in the group receiving co-treatment with FCV and either chloroquine (CQ) or rapamycin (Rapa). This observation was consistent with the Western blot results shown in Figure 3A,B, indicating the induction of autophagy by FCV infection. Conversely, the group treated with FCV and LY294002 exhibited a significant inhibition of GFP-LC3 punctate aggregate generation, as shown in Figure 4, suggesting the involvement of the PI3K/Akt/mTOR signaling pathway in the modulation of autophagy by FCV infection.

### 2.4. Feline Calicivirus P39 Promotes Autophagy and Degrades RIG-I

**Figure 5:**
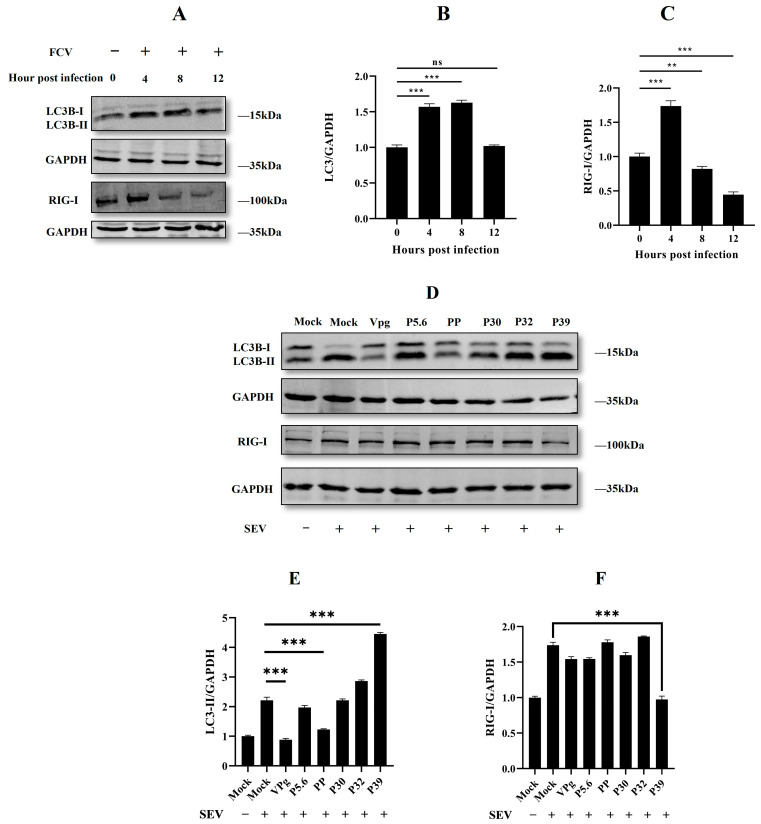
P39 induced autophagy and repressed RIG-I expression. (**A**) CRFK cells were infected with FCV-SCAU-10 at MOI of 1 at 0, 4, 8, and 12 h.p.i. (**B**,**C**) Protein band intensity of LC3B-II, RIG-I, and GAPDH was used for Western blotting quantification analysis. Samples were analyzed in three independent experiments. (**D**) HEK 293T cells were transfected with different plasmids; 24 h post-transfection, cells were stimulated with SEV. At 12 h post-stimulation, WB analysis was performed. (**E**,**F**) Protein band intensity of LC3B-II, RIG-II, and GAPDH was used for Western blotting quantification analysis. (ns—not significant, ** *p* < 0.01, *** *p* < 0.001).

Our lab and a recent study have reported that the innate immune-associated molecules, RIG-I, could be degraded by autophagy [20,21]. Additionally, RIG-I is an important sensor for RNA viruses to trigger the interferon response. Several studies have reported that FCV P30 and P39 suppress the host innate immune response [14,22]. According to this evidence, we were curious about the relationship among feline RIG-I, FCV viral proteins, and autophagy. To address this, we first used Western blotting to determine the autophagy level and the endogenous expression of feline RIG-I in FCV infection. The results showed that both LC3B-II transformation and feline RIG-I were significantly up-regulated 4 h post-infection. However, the protein level of feline RIG-I was quickly reduced 8 and 12 h post-infection. LC3B-II was still remained at a high level 8 h post-infection, while there was no significance 12 h post-infection (Figure 5A). These results suggested a potential time-dependent manner between the feline RIG-I protein and FCV infection-induced autophagy.

To further investigate which viral protein could be related to this manner, we established a plasmid overexpression model followed by Sendai virus (SEV) infection. A previous report has indicated that SEV could promote the autophagy process [23,24]. So, we chose it as the mimic for FCV infection-induced autophagy to ensure simple factor control. The Western blotting results proved the successful establishment of our model. In a further analysis, we found that the overexpression of the FCV protein P39 significantly up-regulated the ratio of LC3B-II/GADPH compared to the only SEV infection group. Surprisingly, we also found that SEV-induced feline RIG-I was significantly reduced under the combination of the overexpression of the FCV protein P39 and SEV infection, while other groups showed no significant change (Figure 5D).

It is also noteworthy that the expression of LC3-II was reduced by VPg and PP. As previously reported, PP has been shown to induce host gene shutoff by promoting the degradation of host mRNAs [15], which presents a potential mechanism by which the immune response is inhibited. It is conceivable that cellular autophagy, which is a part of the innate immune response [25], was suppressed by PP. However, the exact mechanisms by which VPg inhibits cellular autophagy remain largely unknown. One possibility is that VPg interferes with the function of LC3 or other autophagy-related proteins, but this hypothesis requires further investigation.

**Figure 6:**
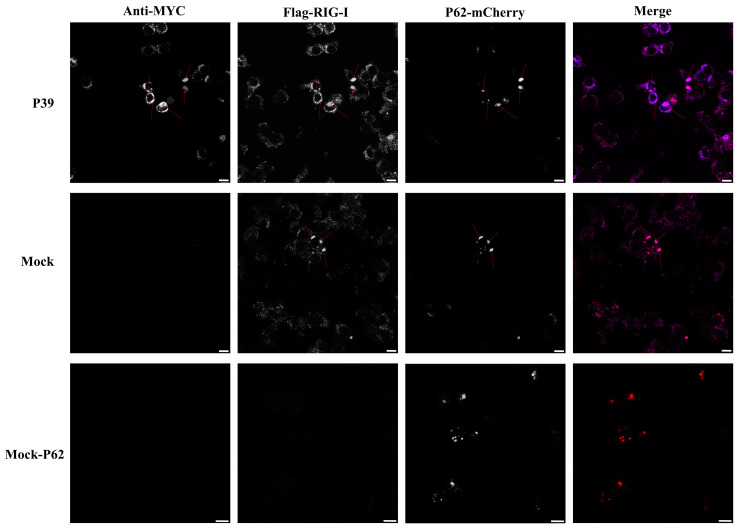
Co-localization of RIG-I, p39, and p62 in HEK-293T cells. HEK-293T cells were co-transfected with p39-myc, RIG-I-Flag, and p62-mCherry plasmids. Cells were stained for Myc (blue), flag (purple), and mCherry (red), and analyzed by confocal microscopy. Scale bar 10 μm.

Two key autophagy receptors, P62 and CCDC50, are involved in the selective removal of damaged proteins and organelles from the cell, but they exhibit different substrate specificities [26,27]. While P62 plays a crucial role in the selective autophagy of ubiquitinated substrates, CCDC50 is involved in the selective autophagy of non-ubiquitinated substrates. Our confocal microscopy analysis revealed a significant co-localization of the innate immune sensor RIG-I and the autophagy receptor P62-mCherry, which is consistent with previous studies [28]. As shown in Figure 6, surprisingly, we also observed a triple-protein co-localization of RIG-I, P62, and the non-structural protein P39-MYC. This suggests a potential interaction between P39 and RIG-I, which may promote RIG-I degradation through the autophagy receptor P62. However, it is important to acknowledge the limitations of our study, which are primarily due to the unavailability of necessary reagents for a further validation of our observations. Despite these limitations, our observations provide a promising direction for a further investigation and understanding of the molecular mechanisms underlying FCV protein–host cell interactions.

**Figure 7:**
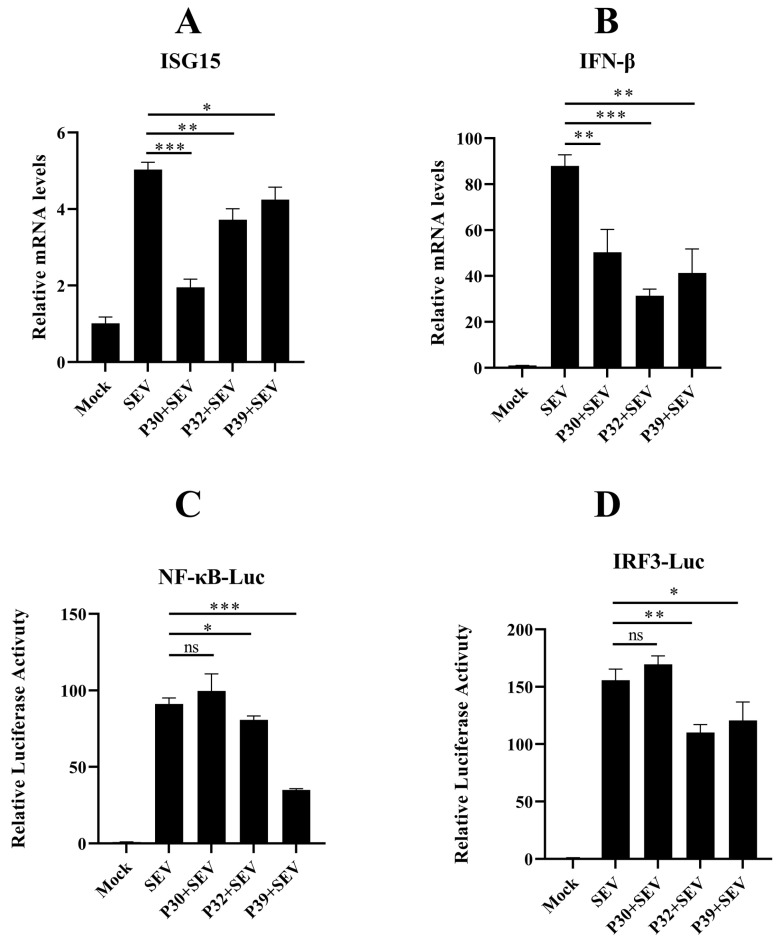
P30, P32, and P39 inhibit innate immune response. (**A**,**B**) HEK 293T cells were transfected with different plasmids, total RNA was extracted from the cells at the indicated time points, and the mRNA levels of IFN-β and ISG15 were measured by qPCR. (**C**,**D**) In the dual luciferase assay, CRFK cells were transfected with different plasmids for 24 h, inoculated with SeV and incubated for another 12 h, and lysed for determination of luciferase activity. P30, P32, and P39 inhibited SeV-activated IFN-β and ISG15 expression at the mRNA level. Second, the results of the dual luciferase assay showed that P32 and P39 blocked the activation of the NF-κB and IRF3 promoters. (ns—not significant, * *p* < 0.05, ** *p* < 0.01, *** *p* < 0.001).

To clarify the relationship between FCV infection and the innate immune response in a more comprehensive manner, we subsequently carried out the qPCR and luciferase assay to evaluate the interferon response. The results indicated that the FCV proteins P30, P32, and P39 significantly suppressed the mRNA level of ISG15 and interferon beta (Figure 7A,B). Moreover, the activity of the NF-κB and IRF-3 promoter were also examined, and the results showed that the FCV proteins P32 and P39 could strongly decrease the SEV-induced activation (Figure 7C,D).

## 3. Discussion

According to previous studies of the virus, autophagy usually plays a dual role in viral infections. On the one hand, autophagy is capable of degrading virus particles in a xenophagy manner, such as in the way that autolysosomes could degrade the influenza A virus (IAV) and human immunodeficiency virus 1 [29,30]. In contrast, many studies have shown that viruses can promote replication by hijacking autophagy. For example, the hepatitis C virus and IAV can block the complete autophagic flow by inhibiting the fusion between autophagic vesicles and lysosomes to promote viral replication [31,32,33,34]. Various pieces of research have revealed the intricate and dualistic relationship between host autophagy and the virus.

Our study investigated the potential relationship between FCV infection and autophagy by examining the induction of autophagosome formation and the recruitment of LC3B to autophagosomal membranes during FCV infection. Specifically, we found that the non-structural proteins P30, P32, and P39 of FCV played a role in the process of autophagosome formation. Although our findings suggest that FCV infection can activate autophagy, further research is needed to fully understand the relationship between FCV viral proteins and other autophagy-related components. Therefore, our results provide preliminary evidence for the activation of autophagy during FCV infection and warrant future investigation into the underlying mechanisms involved.

The complete autophagy process can be divided into two stages: the early stage of autophagy initiation, membrane elongation, and autophagosome maturation, and the late stage of autophagosome–lysosome fusion and autolysosome degradation. In this study, we aimed to investigate the role of the complete autophagy process in FCV replication. To define the precise role of autophagy in FCV replication, we used an autophagy inducer and inhibitor drugs. The results showed that the autophagy inducer (rapamycin) significantly increased the mRNA level of FCV and FCV titer, while the inhibitor of early autophagy (ly294002) dampened FCV replication. These results indicated that the early stage of autophagy initiation, membrane elongation, and autophagosome maturation could promote FCV replication. Despite the fact that our study focused on the complete autophagy process, it is possible that an intermediate step between inhibition by ly294002 and the end of inhibition by chloroquine could positively affect FCV growth. Further studies are needed to investigate this possibility. 

It is known that the endoplasmic reticulum provides the primary biological membrane resource of the autophagosome [35,36]. An earlier report indicated that P30, P32, and P39 localize to the endoplasmic reticulum to initiate replication of the complex formation [10]. The combined consideration of these previous studies and our experimental results suggested that the FCV non-structural proteins P30, P32, and P39 might use the autophagy-related components of the endoplasmic reticulum to modulate the replication complex to potentially facilitate the replication of the virus. 

The unexpected observation that chloroquine, an inhibitor of late autophagy, significantly increased FCV replication indicates that the late autophagic degradation could clear FCV virions. A pre-treatment of different autophagy-related drugs in FCV-infected cells further suggests that FCV infection-induced autophagy may have a complex character, acting as a double-edged sword in different stages.

Many studies have proved that calicivirus infection triggers the host innate immune response and that calicivirus has tactical counteractions. The dsRNA of murine norovirus (MNV) was recognized by the host MDA5, and MDA5 mediates the interferon response [37]. The replication of human norovirus (HuNoV) RNA is sensitive to RIG-I and MDA5, which are potent inhibitors for HuNoV infection [38]. In addition, rabbit hemorrhagic disease virus infection activates interferon response factors [39]. As with other calicivirus infections, feline caliciviruses have developed different strategies to evade and counteract the host innate immune response. The FCV strains F9, HRB-SS, and Bolin fail to trigger the response of IFN-β. FCV P39 suppresses the expression of IFN-β and ISG15 and the phosphorylation of IRF-3 [22]. The FCV strain 2280 P30 antagonizes the interferon response by directly degrading IFNAR1 mRNA [14].

Autophagy has been reported as the regulator of the antiviral response. It has been shown that the Atg5–Atg12 conjugate directly associates with RIG-I through the caspase recruitment domains (CARDs) to block innate antiviral immune responses and, consequently, facilitate viral replication [40]. RIG-I-mediated signaling is capable of triggering autophagy through the MAVS-TRAF6-Beclin1 signaling axis [19]. In calicivirus infection, MNV infection activates the IFN-γ-mediated antiviral effect, which requires the ATG5-ATG12-ATG16L1 autophagic complex [41]. Our lab has proved that feline RIG-I plays an essential role in the antiviral effect of FCV infection (not shown in this study). Moreover, our previous study indicated that autophagy could negatively regulate RIG-I [20]. In this study, we found that FCV infection triggered autophagy. Thus, these results sparked our curiosity regarding the relationship between feline RIG-I and FCV infection-induced autophagy. The quantification of the protein expression feline RIG-I in different time points showed that feline RIG-I was activated by FCV infection 4 h post-infection, and it was, subsequently, significantly inhibited 8 h and 12 h post-infection. Meanwhile, a high level of LC3B lipidation was detected 4 h and 8 h post-infection. To further explore whether FCV infection-induced autophagy was involved in the negative regulation of feline RIG-I, the SEV was used to mimic the FCV infection. The Western blotting results of HEK293T cells overexpressing FCV non-structural proteins, followed by SEV infection, showed that only FCV P39 could up-regulate the SEV-induced LC3B lipidation and reduce the protein expression of feline RIG-I. Additionally, a further qPCR and luciferase assay also proved the ability of FCV P39 to inhibit the innate immune response. These findings resulted in us speculating that the autophagy-related machinery of FCV P39 mediated the suppression of feline RIG-I.

This study provides a foundation for understanding the role of FCV infection-induced autophagy by revealing a novel mechanism of the FCV P39-mediated suppression of feline innate immunity. However, further research is needed to explore the interactions between the non-structural proteins (P30, P32, and P39) of FCV, host autophagy, and the host innate immune response. Such research could help elucidate the mechanism of the FCV replication cycle and potentially lead to the development of autophagy-targeted drugs to inhibit or prevent FCV infection.

## 4. Materials and Methods

### 4.1. Cells, Virus, Plasmid, Reagents, and Antibodies

The human embryonic kidney (HEK) 293T cell line (CRL-3216) and Crandell Reese feline kidney (CRFK) cell line (CCL-94) was purchased from ATCC in 2014; it was appropriately cryopreserved, cultured, and passaged in our lab. CRFK cells and HEK 293T cells were cultured in DMEM medium (DMEM; Biological Industries, Kibbutz Beit-Haemek, Israel), supplemented with 10% fetal bovine serum (Biological Industries) and 1% penicillin/streptomycin, and cultured in 5% CO_2_ at 37 °C.

The FCV-SCAU-10 strain of feline calicivirus (FCV) was propagated in CRFK cells. The plasmids used in this research are listed in Supplementary Table S1. The antibodies used in this study are listed in Supplementary Table S2. 

### 4.2. Cell Seeding, Transfection, RNA Extraction, and cDNA Synthesis

The experimental procedure was performed as follows: the cells were seeded into 6-well plates and allowed to reach 80% confluence. Transfection was performed using lipo8000 (Beyotime, Shanghai, China), and each well was transfected with a total of 2000 ng of plasmid DNA. This transfection protocol was selected based on preliminary experiments to determine the optimal transfection conditions for our specific cell line and plasmid constructs. The total RNA was extracted from the cells with a Simply P Total RNA Extraction Kit (Bioer Technology, Hangzhou, China) according to the manufacturer’s instructions. The RNA samples were immediately stored at −80 °C. Then, cDNA was made from 1000 ng of total RNA using the HiScript III 1st Strand cDNA Synthesis Kit (Vazyme, Nanjing, China) and they were used for qPCR.

### 4.3. Fluorescence Analysis

To determine the subcellular localization of LC3 proteins, when HEK293T cells reached 60% confluence, and the cells were transfected with myc-P30, myc-P32, and myc-P39 plasmid along with LC3-GFP plasmid, after 24 h, the cells were fixed with 4% cold paraformaldehyde (Biosharp, Hefei, China) for 10 min, were washed 3 times with PBS, and blocked with QuickBlock Blocking Buffer (Beyotime, Shanghai, China) for 10 min at room temperature, and they were then incubated with a primary antibody diluted with Immunol Staining Primary Antibody Dilution Buffer (Beyotime, Shanghai, China) for 1 h at 37 °C. The cells were washed 3 times and incubated with a corresponding fluorescently labeled secondary antibody for 1 h at 37 °C in the dark. After the cells were washed 3 times with PBS, the nuclei were stained with DAPI (Beyotime) for 5 min. The fluorescence signal was observed with the confocal laser scanning microscope Leica SP8 TCS (Leica, Wetzlar, Germany).

### 4.4. Luciferase Assay

To analyze the effect of three viral proteins on the IFN-β and NF-κB response, HEK293T cells (5 × 10^4^ cells/well) were co-transfected with 0.5 μg of the reporter plasmid and 0.02 μg of the pRL-TK plasmid, along with myc-P30, myc-P32, and myc-P39 expression plasmid and empty plasmid (as the control). After 12 h, the cells were lysed and the luciferase activity in the lysates was measured with the Dual-Luciferase Reporter Assay Kit (Vazyme) according to the manufacturer’s instructions. The relative luciferase activity (RLA) in each sample was calculated as the ratio of the firefly luciferase activity to the Renilla luciferase activity.

### 4.5. Western Blotting

The cells were washed with precooling phosphate-buffered saline (PBS) three times and lysed on ice with Western and IP Lysate buffer (Beyotime) containing 1% protease inhibitors (Selleck Chemicals, Shanghai, China). Lysates were collected and centrifuged at 15,000 rpm for 15 min, were subjected to sodium dodecyl sulfate–polyacrylamide gel electrophoresis (SDS–PAGE), and were finally transferred to polyvinylidene fluoride (PVDF) membranes (Millipore, Burlington, MA, USA). The membranes were blocked for 1 h at room temperature with 5% skim milk powder in PBS; they were then incubated overnight at 4 °C with primary antibodies. After the membranes were washed with PBS containing 0.1% Tween 20 (0.1% PBST), the corresponding secondary antibodies were incubated for 1 h at room temperature. Protein bands were visualized by Odyssey Sa (Li-cor, Lincoln, NE, USA). The protein bands were quantified with the ImageJ software v1.51j8 (National Institutes of Health, Bethesda, MD, USA).

### 4.6. Real-Time qPCR Analysis

The standard curve method was employed for virus quantification. A pair of specific qPCR primers were designed based on the FCV-SCAU-10 sequence. The amplified fragment was ligated into the TA/Blunt-Zero cloning vector (Vazyme, Nanjing, China), then gradient dilution (10^1^~10^9^ copies/μL) of the plasmids was used to construct a standard curve. Afterward, the mRNA levels of FCV were calculated by the established standard curve. For the reverse transcription real-time qPCR analysis, the viral RNA was extracted with the Simply P Total RNA Extraction Kit. The cDNA was generated with the HiScript III 1st Strand cDNA Synthesis Kit (Vazyme) and analyzed by qPCR using the ChamQ SYBR Color qPCR Master Mix (Vazyme, Nanjing, China), according to the manufacturer’s protocol. The viral titers were calculated according to the standard curve.

To detect changes in the expression of interferon-related genes in the cells, the mRNA expression levels of IFN-β and ISG15 were determined, and GAPDH was used as the housekeeping gene. The primers used are listed in Supplementary Table S3. The relative mRNA expression of each target gene was calculated using the 2^−ΔΔCt^ method.

### 4.7. Transmission Electron Microscopy

CRFK cells were seeded into the 6-well cell plate. The monolayer cells were infected with FCV. Twelve hours post-infection, the cells were collected by centrifugation. The cell masses were fixed by a fixer for electron microscopy (Servicebio, Wuhan, China). After the fixation, the following procedure was performed for the dehydration: embedding, curing, block repair, sectioning, and staining. The dried sections were observed under the Talos L120C TEM (Thermo Fisher Scientific, Waltham, MA, USA).

### 4.8. Autophagy-Related Treatment

CRFK cells were seeded in 6-well plates. The cells were transfected with the plasmids after reaching 80% confluence. Subsequently, after transfection for 24 h, the cells were treated with rapamycin (0.5 µM, acting as an autophagy inducer), ly294002 (10 µM, acting as an autophagy inhibitor), and chloroquine (25 µM, acting as an autophagy inhibitor) for 12 h.

### 4.9. Statistical Analysis

All data were analyzed by the unpaired Student’s *t*-test using Prism v9.3 (GraphPad Software, San Diego, CA, USA) (mean ± SD, * *p* < 0.05, ** *p* < 0.01, *** *p* < 0.001).

## 5. Conclusions

In conclusion, our study has provided evidence that FCV infection can induce cellular autophagy through the actions of its non-structural proteins P30, P32, and P39. We have also demonstrated that autophagy plays a vital role in the FCV replication cycle. Additionally, we have discovered that the non-structural protein P39 employs the autophagic pathway to inhibit the innate immune response by degrading the innate immune protein RIG-I. These findings collectively highlight the multifaceted interplay between FCV and cellular autophagy, and shed light on the intricate mechanisms by which FCV evades host defenses.

## Data Availability

The data that support the findings of this study are available from the corresponding author upon reasonable request.

## Supplementary Materials

The following supporting information can be downloaded at: https://www.mdpi.com/article/10.3390/ijms24065254/s1.
